# Different screening frequencies of carbapenem-resistant Enterobacteriaceae in patients undergoing hematopoietic stem cell transplantation: which one is better?

**DOI:** 10.1186/s13756-020-0706-0

**Published:** 2020-03-17

**Authors:** Ting-Ting Yang, Xue-Ping Luo, Qing Yang, Hong-Chao Chen, Yi Luo, Yan-Min Zhao, Yi-Shan Ye, Xiao-Yu Lai, Jian Yu, Ya-Min Tan, Guo-Qing Wei, He Huang, Ji-Min Shi

**Affiliations:** 1 Bone Marrow Transplantation Center, the First Affiliated Hospital, Zhejiang University School of Medicine, 79 Qingchun Road, Hangzhou, 310003 China; 2 Institute of Hematology, Zhejiang University, Hangzhou, China; 3 Zhejiang Engineering Laboratory for Stem Cell and Immunotherapy, Hangzhou, China; 4 Department of Laboratory Medicine, the First Affiliated Hospital, Zhejiang University School of Medicine, Hangzhou, China; 5 Key Laboratory of Clinical In Vitro Diagnostic Techniques of Zhejiang Province, 79 Qingchun Road, Hangzhou, China

**Keywords:** Carbapenem-resistant Enterobacteriaceae, Hematopoietic stem cell transplantation, Continuous screening, Bloodstream infection

## Abstract

**Background:**

A consensus has been reached that carbapenem-resistant *Enterobacteriaceae* (CRE) screening in immunosuppressed individuals can reduce the incidence of CRE bloodstream infection (BSI).

**Methods:**

We retrospectively studied the clinical data of 395 consecutive HSCT patients from September 2017 to April 2019. From September 2017 to June 2018 (period 1), 200 patients received single CRE screening before transplantation. From July 2018 to April 2019 (period 2), 195 patients received continuous weekly CRE screening after admission. For patients colonized with CRE, targeted managements were received: (1) contact precautions and (2) preemptive CRE-targeted treatment if necessary.

**Results:**

During period 1, 3 patients with CRE colonization were detected (1.5%). The CRE BSI rate was 2.0% (4 patients), and the related 30-day mortality was 50.0% (2 out of 4 patients). During period 2, 21 patients with CRE colonization were detected, and the detection rate was significantly higher than that in period 1 (*P* < 0.001). Of the 21 colonized patients, 4 (19.0%) patients were identified as positive for CRE at the first screening, 5 (23.8%) were identified at the second screening, and the remaining 12 (57.1%) were identified at the third or later screening. The CRE BSI rate decreased to 0.5% (1/195), and there were no CRE-related death.

Fifteen colonized patients developed neutropenic fever. Thirteen colonizers were preemptively treated with tigecycline within 24 h of fever onset, and they achieved rapid temperature control. One colonizer received tigecycline later than 48 h after fever onset and ultimately survived due to the addition of polymyxin. The other received tigecycline later than 72 h after fever onset and died of septic shock.

**Conclusion:**

The increase in screening frequency contributed to the detection of patients with CRE colonization. Targeted managements for these colonized patients may contribute to reducing the incidence and mortality of CRE BSI, therefore improving the prognosis of patients.

## Introduction

With the extensive use of carbapenems, a global dissemination of carbapenem-resistant *Enterobacteriaceae* (CRE) has been reported in recent decades [1]. A recent national multicenter study from 25 provinces in China reported that the rate of carbapenem resistance in *Escherichia coli* (*E. coli*) and *Klebsiella pneumoniae* (*K. pneumoniae*) was up to 0.6–3.6% and 1.2–18.9%, with 1.8 and 12.3% in Zhejiang Province, respectively, which has increased year after year [2]. Long-term hospitalization, frequent use of broad-spectrum antibiotics, high-dose chemotherapy, neutropenia, compromised immunity, and gastrointestinal mucosal destruction are risk factors favoring CRE colonization and bloodstream infections (BSIs) in patients with hematologic malignancies undergoing hematopoietic stem cell transplantation (HSCT) [3–8]. The overall incidence of BSIs caused by CRE in HSCT recipients is approximately 1.8–2%. Due to the lack of effective antibiotic treatment, the death rate of CRE BSIs after HSCT is high (51–65%) [3, 6, 9, 10]. Increasing studies have revealed the importance of preemptive intervention in the prevention and treatment of CRE BSIs.

Gut colonization by CRE is an independent risk factor for CRE infection [7, 8, 11, 12]. An Italian multicenter study showed that in patients who underwent autologous and allogeneic HSCT, the CRE colonization rates were 1 and 2.4%, respectively. Of these patients with CRE colonization, 25.8 and 39.2% subsequently developed CRE BSIs, which was significantly more frequent than the rate of general hospitalized patients (16.5%) [6]. Early identification of CRE colonization made it easier to adopt early strategies to control CRE dissemination [13]. Many guidelines and studies also recommend HSCT patients as a target population for CRE screening, and consider stool as the preferred sample for screening because of good patient compliance and few side effects [5, 14–18]. However, for HSCT centers where CRE are endemic nosocomial pathogens, the effect of weekly active screening on morbidity and mortality of CRE BSIs has rarely been reported.

In our center, the first case of CRE BSI was identified in May 2016, and the incidence and related mortality of CRE BSIs from May 2016 to August 2017 was 1.9 and 66.7%, respectively. Given the high CRE BSI-related mortality, we initiated active screening from September 2017 in an attempt to identify CRE-colonized patients and to reduce the rate of CRE BSIs. This study aimed to evaluate the effect of CRE screening and intervention measures in the prevention and control of CRE BSI during the early stage of transplantation.

## Patients and methods

### Patients and study design

This was a retrospective observational study of 395 consecutive patients who underwent HSCT in our center from September 2017 to April 2019. From September 2017 to June 2018 (period 1), we implemented single CRE rectal screening within 1 week before transplantation. From July 2018 to April 2019 (period 2), to improve the positive detection rate, we implemented continuous weekly screening until hematopoietic reconstruction. CRE screening would be repeated in patients who had fever or underwent gut complications, such as abdominal pain, diarrhea and perianal inflammation. Data retrospectively collected from the two periods were compared. The CRE gut colonization rate, CRE BSI rate and attributed mortality at day 30 from the positivity of blood cultures were evaluated. Informed consent for HSCT, collection of stool swabs, data analysis, and publication was obtained from patients. The study protocol complied with the Declaration of Helsinki.

### Surveillance

Stool was the surveillance material in our center. Specimens were collected in an aseptic manner and immediately transported to the microbiology laboratory. The technician inoculated the samples on Columbia blood agar for microbial identification, and antibiotic susceptibility testing was performed. CRE was defined as any isolated *Enterobacteriaceae* resistant to meropenem (disc diffusion diameter ≤ 19 mm), ertapenem (MIC≥2 μg/ml) or imipenem (MIC≥4 μg/ml) [19]. Patients who had a positive stool swab for CRE during the screening period were defined as colonized, while patients who continuously tested negative for CRE during the whole screening period were defined as noncolonized [14].

### Targeted managements of CRE colonization

For patients colonized with CRE, contact precautions were applied, including a single room, a hanging sign, hand hygiene performed before and after entering the room, use of disposable gloves and gowns and strengthened environmental cleaning and disinfection. Patients who were colonized with CRE but had no fever also received contact precaution but were not treated with prophylactic antibiotics.

A single oral temperature of ≥38.3 °C or a persistent oral temperature > 38.0 °C sustained over 1 h was defined as fever, and an absolute neutrophil count (ANC) below 0.5 × 10^9^/L was defined as neutropenia in this study [20]. CRE BSI was diagnosed with the collection of blood cultures that yielded a CRE strain. When the colonized patient exhibited febrile episodes, treatment was conducted according to the patient’s condition, neutropenic severity, infection clinical symptoms and laboratory examination. At least two consecutive blood cultures were sent before starting antibiotics for patients with clinical symptoms. Preemptive CRE-targeted treatment with tigecycline was performed under all the following conditions: (i) CRE colonization identified at the onset of fever; (ii) neutropenic fever persistent over 12 h or fever with obvious symptoms of intestinal infection, such as abdominal pain, diarrhea, and perianal pain; and (iii) C-reactive protein (CRP) at 5 times higher than normal with or without a significant increase in procalcitonin (PCT). Polymyxin was added if there were signs of progression of sepsis or if there was a lack of response for tigecycline treatment within 48 h [18, 21].

### Statistical analysis

Data analyses were performed with SPSS (version 20.0 SPSS Inc., IBM Co., Chicago, IL, USA). Continuous variables were evaluated using the Kruskal-Wallis test; categorical variables were evaluated with Fisher’s exact test or Pearson chi square test. All tests and *P*-values were two-sided, and a *P*-value < 0.05 was considered statistically significant.

## Results

### Patient characteristics

A total of 395 patients who underwent HSCT from September 2017 to April 2019 were evaluated. Two hundred and twenty-one (55.9%) were male, and 174 (44.1%) were female, with a median age of 36 years (range, 9–67). Table 1 shows the demographic and clinical characteristics of the patients. There was no significant difference between the two groups in age, sex, underlying disease or type of HSCT.

**Table 1:** Baseline demographic and clinical characteristics of the study population

Variable	Single screening group (*N* = 200)	Continuous screening group (*N* = 195)	*P* Value
Age, median (range)	35.7(9–64)	37.2(12–67)	0.319
Sex, N (%)			0.530
Male	115(57.5)	106(54.4)	
Female	85(42.5)	89(45.6)	
Underlying disease, N (%)			0.208
AML	78(39.0)	66(33.8)	
ALL	62(31.0)	66(33.8)	
MDS	18(9.0)	9(4.6)	
NHL/HL	21(10.5)	20(10.3)	
MM	12(6.0)	20(10.3)	
others	9(4.5)	14(7.2)	
Type of HSCT, N (%)			0.697
Allogeneic			
MUD	13(6.5)	14(7.2)	
Haplo	127(63.5)	117(60.0)	
Sib	37(18.5)	34(17.4)	
Autologous	23(11.5)	30(15.4)	

*Abbreviations*: *AML* Acute myeloid leukemia, *ALL* Acute lymphatic leukemia, *MDS* Myelodysplastic syndrome, *NHL* Non-Hodgkin lymphoma, *HL* Hodgkin lymphoma, *MM* Multiple myeloma, *MUD* Matched-unrelated donor allogeneic HSCT, *Haplo* HLA-Haploidentical allogeneic HSCT, *Sib* HLA-sibling allogeneic HSCT

### CRE detection rate, BSI incidence and clinical outcomes

During period 1, 3 patients (3/200, 1.5%) were identified as colonized with CRE in the gut; one died of septic shock, and the other two survived. Four patients who were screened negative for CRE colonization before HSCT later developed CRE BSIs (4/200, 2%) during the HSCT period. Two (50%) out of the 4 patients died, as one patient died of CRE-related septic shock and the other of CRE BSI-induced thrombotic microangiopathy. During period 2, 21 (10.8%) out of 195 patients were identified to be colonized with CRE, which was a significantly higher percentage than that identified by single screening (*p* < 0.001). Among these patients, the median number of screening times performed in patients was 6 (ranging from 4 to 15 times): only 4 (19.0%) patients were identified as positive for CRE at the first screening, 5 (23.8%) at the second screening, and the remaining 12 (57.1%) at the third or more screening. One of these colonized patients with neutropenia subsequently developed CRE BSI (1/21, 4.8%) but survived (Table 2).

**Table 2:** Implementation rate and results of CRE screening

	Without screening (*N* = 311)	Single screening (period 1, *n* = 200)	Continuous screening (period2, *n* = 195)
Patients diagnosed as CRE colonization, N (%)	/	3(1.5%)	21(10.8%)
CRE BSIs, N (%)	6 (1.9%)	4(2.0%)	1(0.5%)
patients with CRE colonization	0	4	0
patients without CRE colonization	6	0	1
Mortality in patients with CRE BSI, N (%)	4 (66.7%)	2(50.0%)	0

### Targeted managements

Twenty-four patients (3 detected during period 1 and 21 detected during period 2) were identified to be colonized with CRE in the gut, and the clinical manifestations are summarized in Table 3. Among the 3 patients who received a single screening, 2 patients were positive for CRE screening before transplantation and developed fever at day + 7 and day + 5 after screening. They received tigecycline-based treatment within 24 h of developing fever, and their body temperatures were controlled within 72 h of fever onset. The other patient (patient 1) was negative for CRE screening before HSCT. However, due to symptoms of persistent neutropenic fever, abdominal pain, diarrhea and significantly increased CRP (over 100 mg/L) after HSCT, the stool swab of this patient was sent for another CRE screening, which indicated positive CRE colonization at 3 days after fever initiation. She was then treated with tigecycline but ultimately died of septic shock.

**Table 3:** Clinical manifestations of the 24 patients colonized with CRE

Pt	Age /Sex	Disease/HSCT	CRE Isolate	Which times screening turned positive	Febrile episodes in carriers	Time from colonization to fever (day)	Time from fever to the use of tigecycline (hour)	ANC at fever (×10^9^/L)	Symptoms at fever	CRP (mg/L) /PCT (ug/L)	30 days status
Single screening											
1^a^	36/F	ALL/Haplo	*E.coli*	/	Yes	−3	> 72	0	Abdominal pain, diarrhoea	216.8/1.0	Dead
2	56/F	MM/Auto	*E. coli*	1	Yes	7	< 24	0	Chills, tremble	95.1/4.1	Alive
3	32/m	ALL/MUD	*K.pneumoniae*	1	Yes	5	< 24	0	No	48.9/normal	Alive
Continuous screening											
4	36/M	AML/Haplo	*K.pneumoniae*	5	No	/	/	/	/	/	Alive
5	15/M	AML/Haplo	*E. coli*	2	Yes	9	< 24	0	Chills, tremble	73.0/normal	Alive
6	27/M	ALL/Haplo	*E. coli*	3	Yes	8	< 24	0	No	52.2/normal	Alive
7	48/M	AML/Haplo	*E.coli*	2	Yes	4	< 24	0	Perianal inflammation	112.3/ normal	Alive
8	45/M	AML/Haplo	*K.pneumoniae*	4	No	/	/	/	/	/	Alive
9	40/M	AL/Haplo	*K.pneumoniae*	1	No	/	/	/	/	/	Alive
10	38/M	T-LBL/Haplo	*E. coli*	3	Yes	11	< 24	0.5	No	43.2/normal	Alive
11	16/F	ALL/Haplo	*E. coli*	1	Yes	17	< 24	0	No	54.2/normal	Alive
12	18/M	ALL/MUD	*E.coli*	2	Yes	9	< 24	0	Chills, tremble	64.5/normal	Alive
13	25/M	ALL/Haplo	*E.coli*	2	Yes	8	< 24	0	No	61.2/normal	Alive
14	34/F	AML/Haplo	*K.pneumoniae*	3	No	/	/	/	/	/	Alive
15	44/M	MM/Auto	*E.coli*	4	No	/	/	/	/	/	Alive
16	50/M	AML/Sib	*K.pneumoniae*	1	Yes	15	< 24	0	sore throat	70.4/normal	Alive
17	18/M	ALL/Haplo	*K.pneumoniae*	5	No	/	/	/	/	/	Alive
18	51/M	ALL/Haplo	*E.coli*	5	No	/	/	/	/	/	Alive
19	22/M	ALL/Haplo	*K.pneumoniae*	4	No	/	/	/	/	/	Alive
20	22/F	AML/Haplo	*K.pneumoniae*	3	Yes	1	> 48	0	Chills, tremble	65.2/0.6	Alive
21	26/M	ALL/MUD	*K.pneumoniae*	1	Yes	13	< 24	0	No	73.4/normal	Alive
22	27/M	ALL/Haplo	*K.pneumoniae*	6	No	/	/	/	/	/	Alive
23	21/M	AML/Haplo	*K.pneumoniae*	2	Yes	12	< 24	0	Perianal inflammation	307/ normal	Alive
24	62/M	AML/Haplo	*K.pneumoniae*	6	Yes	2	< 24	0	No	141.7/normal	Alive

*Abbreviations*: *AML* Acute myeloid leukemia, *ALL* Acute lymphatic leukemia, *AL* Acute leukemia, *Auto* Autologous HSCT, *CRP* C-reactive protein (normal value: 0-8 mg/L)*E.coli Escherichia coli, Haplo* HLA-Haploidentical allogeneic HSCT, *K.pneumoniae Klebsiella pneumoniae, MM* Multiple myeloma, *MUD* Matched-unrelated donor allogeneic HSCT, *PCT* Procalcitonin (normal value:< 0.5μg/L), *Sib* HLA-sibling allogeneic HSCT, *T-LBL* T-lymphoblastic lymphoma

^a^The patient 1:single screening was negative for the patient, but subsequent screening indicated positive 3 days after the onset of fever and the patient died of septic shock

1.Four patients (2,12,20,23) were combined with polymyxin because of persistent fever after 48 h of tigecycline treatment, one of them (patient 20) developed CRE BSIs later

During period 2, febrile episodes did not occur after positive screening in 9 patients, and antibiotics were not used. For the other 12 colonized patients, they developed neutropenic fever, and the median time from colonization identification to fever was 9 days (1–17 days). They received treatment with tigecycline in the initial 24 h of following fever onset (except patients 20 received tigecycline after 48 h of fever onset) considering the emergence of neutropenic fever, accompanying clinical signs of infection and significantly increased inflammatory biomarkers: the body temperatures of 9 patients were controlled within 72 h of fever onset. Three patients (patients 12, 20, and 23) continued to suffer high fever (over 38.5 °C) 48 h after tigecycline treatment and were additionally treated with polymyxin. Polymyxin was changed to ceftazidime-avibactam due to the nephrotoxicity in patient 20. The patient 20 was diagnosed CRE BSI 3 days after fever. The body temperatures of 3 patients returned to normal after the administration of targeted treatment. Finally, 21 colonized patients were discharged after successful hematopoietic reconstruction.

### Microbiological data

CRE strains were isolated from 11 patients in blood cultures, including strains detected in the unscreened phase and 24 patients in stool swabs during the screening period (the first positive result was documented for samples from the same origin), revealing 22 *K. pneumoniae* and 13 *E. coli*. *K. pneumoniae* was predominant, accounting for 81.8% of CRE BSIs (9/11) and 54.2% (13/24) of CRE colonization. One patient with CRE colonization subsequently developed a CRE BSI, and the resistance to antibiotics was exactly consistent between blood culture and stool samples. The resistance rates to antibiotics of the 35 CRE isolates are shown in Table 4. Among the three carbapenems (meropenem, imipenem and ertapenem), there was a slight difference in the antibiotic resistance rate of the two strains, which cannot be ruled out for technical reasons. The resistance rates of tigecycline and amikacin were 0 and 28.6%, respectively.

**Table 4:** The Resistance of CRE to antibiotics in transplant patients (%)

Antibiotics	CRE resistance rate in stool samples			CRE resistance rate in blood cultures		
	Total (24)	*K.pneumoniae* (13, 54.2%)	*E.coli* (11, 45.8%)	Total (11)	*k.pneumoniae* (9, 81.8%)	*E.coli* (2, 18.2%)
Ceftazidime	100%	100%	100%	100%	100%	100%
Ceftriaxone	100%	100%	100%	100%	100%	100%
Cefepime	100%	100%	100%	100%	100%	100%
Piperacillin/tazobactam	100%	100%	100%	100%	100%	100%
Fluoroquinolones	95.8%	100%	90.9%	100%	100%	100%
Gentamicin	82.6%	84.6%	80.0%	63.6%	66.7%	50.0%
Amikacin	16.7%	23.1%	9.1%	54.5%	66.7%	0
Aztreonam	79.2%	92.3%	63.6%	100%	100%	100%
Meropenem	100%	100%	100%	83.3%	100%	50.0%
Imipenem	91.6%	92.3%	90.9%	100%	100%	100%
Ertapenem	100%	100%	100%	100%	100%	100%
Tigecycline	0	0	0	0	0	0
SMZ co	91.7%	84.6%	100%	81.8%	77.8%	100%
Nitrofurantin	/	/	NR	77.8%	100%	0
Tobramycin	/	/	NR	77.8%	85.7%	50.0%

*Abbreviations*: *E.coli Escherichia coli*, *K.pneumoniae Klebsiella pneumoniae*, *SMZ co* Compound Sulfamethoxazole

## Discussion

CRE BSIs have become a major challenge and are associated with high mortality. In China, the overall annual CRE incidence is approximately 4 per 10,000 discharges, with the highest rates in some regions (0.15%) where CRE is highly endemic and causes a severe disease burden [22]. Active surveillance for CRE colonization as part of a multifactorial intervention is an effective strategy to decrease nosocomial CRE infection rate and CRE-related mortality [20, 23, 24]. CRE screening with stool is considered an effective method for surveillance of CRE colonization.

Before the implementation of active screening, the incidence and attributable mortality of CRE BSI in our center were high (1.9 and 66.7%, respectively) and similar to those in previous studies. Thus, we initiated active surveillance in HSCT recipients. During period 1, only 1.5% of patients were identified as positive for CRE, and the 4 patients who developed CRE BSIs were negative at the single screening. The incidence and mortality of CRE BSIs were 2 and 50%, respectively. These results suggested that single screening could not effectively discriminate colonized patients. Considering the poor efficiency, we started continuous weekly screening during period 2, and a significant difference was observed with respect to the CRE detection rate (1.5% vs 10.8%, *P* < 0.001). Additionally, it is worth noting that of the patients who were positive in continuous screening, only 4 were positive at the first screening, and 80.9% (17 out of 21 colonized patients) turned positive results for CRE at the second or later screening, which again suggests the deficiencies of single screening. Moreover, due to the increased positive rate of continuous screening and the preemptive treatments used in these patients with infectious symptoms, the morbidity and mortality of CRE BSIs showed a trend of reduction (only 0.5 and 0%, respectively), for which the difference was not statistically significant, probably owing to the small sample size. Forcina et al. also demonstrated that after the introduction of regular surveillance, the cumulative incidence in CRE BSI and septic shock at 1 year after HSCT was significantly reduced [4]. The CRE infection mortality dropped from 62.5 to 16.6%. These results strongly support the key role of continuous screening in the prevention and control of CRE BSI.

Consistent with published data, the resistance rate to tigecycline in our study was low, at 0%, and tigecycline was also used as the first-line antibiotic for CRE infection in our study [2, 25, 26]. Clinical signs of infection are usually attenuated or absent in neutropenic patients, and fever is often the only symptom of a serious potential infection. In order to manage patients colonized with CRE, who are at high risk of developing BSIs, we conducted contact precaution and preemptive CRE targeted treatment. Early targeted antibiotic therapy is considered vital to cover possible infections in febrile neutropenic patients with CRE colonization even if blood cultures remain negative [20, 27–30]. In our study, we administered preemptive treatment with tigecycline in the initial 24 h following fever onset to patients with CRE colonization combined with neutropenic fever/clinical signs of infection/significant elevation of inflammatory markers, and controlled temperature rapidly. However, 2 patients with neutropenic fever received tigecycline later than 24 h after fever onset. One received tigecycline 72 h since the single screening was negative and ultimately died of septic shock. The other subsequently developed a CRE BSI but survived due to timely combination with polymyxin (changed later to ceftazidime-avibactam because of the nephrotoxicity). These results also highlight that for CRE colonized patients with febrile neutropenia and clinical signs of infection, prompt and active targeted CRE treatment contribute to improvement of prognosis [4, 31]. For patients colonized with CRE without fever, contact precaution is necessary, while the administration of antibiotics should be cautious and avoid abuse. In our study, patients colonized with CRE in the absence of febrile episodes were only isolated and observed, did not receive tigecycline treatment and got favorable prognosis. These results also suggest that gut CRE colonization alone is not an indication for further antibiotic treatment.

There are several limitations of this study. First, this was a single-center, retrospective observational study in a particular geographical area. Second, the resistance mechanism has not been well investigated. Finally, the cohort of patients with CRE colonization or CRE BSIs was small. A multicenter, prospective study is being carried out in our center to further demonstrate the optimal surveillance methods for CRE colonization in HSCT patients.

## Conclusion

In summary, for patients who undergo HSCT, regular continuous screening for CRE colonization is necessary to assist in the early detection and management of CRE colonizers, including contact precaution and prompt implementation of CRE-targeted preemptive treatment to improve outcomes in patients after HSCT with CRE BSIs. Regular continuous gut screening is recommended as a feasible and reliable measure for HSCT patients at high risk of infection.

## Data Availability

The datasets used and/or analyzed during the current study are available from the corresponding author on reasonable request.

## Abbreviations

BSI: Bloodstream infection
CRE: Carbapenem-resistant *Enterobacteriaceae*
CRP: C-reactive protein
*E.coli*: *Escherichia coli*
HSCT: Hematopoietic stem cell transplantation
*K. pneumoniae*: *Klebsiella pneumoniae*
PCT: Procalcitonin
